# Structure–Function Relationships in Polysaccharide–Iron Complexes: Molecular Characterization, Acid-Stress Release Stability, and Gastrointestinal Tolerability

**DOI:** 10.3390/pharmaceutics18070896

**Published:** 2026-07-21

**Authors:** Xiangqiu Qi, Hongwei Zhu, Xin Yan, Xi Kang, Dandan Xiao, Xianyi Sha

**Affiliations:** 1 Quzhou Fudan Institute, Quzhou 324002, China; qixq@qzfudan.com (X.Q.); zhuhw@qzfudan.com (H.Z.); 2 School of Pharmacy, Fudan University, Shanghai 201203, China; 24111030090@m.fudan.edu.cn (X.Y.); 22301030095@m.fudan.edu.cn (X.K.); 3 The Second People’s Hospital of Quzhou, Quzhou 324000, China

**Keywords:** polysaccharide–iron complex, non-biological complex drugs, structure–function relationship, acid-stress release, gastrointestinal tolerability, intestinal iron deposition, quality evaluation

## Abstract

**Background**: Polysaccharide–iron complexes (PICs) are widely used oral iron supplements, but their gastrointestinal tolerability varies and remains incompletely understood. As typical non-biological complex drugs (NBCDs), PICs exhibit structural heterogeneity, and their functional performance may be linked to higher-order structural attributes. **Methods**: In this study, two commercial PIC preparations (test samples A and B) were comparatively investigated to explore their structure–function relationship using a multi-dimensional approach. Structural properties were characterized by gel permeation chromatography (GPC), mass spectrometry (MS), Fourier-transform infrared spectroscopy (FTIR) and nuclear magnetic resonance (NMR) spectroscopy, along with monosaccharide composition analysis. Functional behaviors and physiological relevance were further evaluated through in vitro acid-stress release studies and in vivo rat gastrointestinal tolerability assessments. **Results**: The results revealed that test sample A exhibited a glucose-only detectable monosaccharide profile but a higher and broader apparent molecular-weight distribution, indicating monosaccharide compositional uniformity together with macromolecular heterogeneity. In contrast, test sample B showed detectable glucose and mannose, a lower and narrower apparent molecular-weight distribution, higher measured free iron, and greater iron release under the tested acidic conditions. An exploratory 7-day rat gastrointestinal tolerability study (*n* = 4 per group) indicated that these distinct profiles may impact mucosal tolerability. Structurally stable test sample A allowed intestinal iron accumulation while maintaining mucosal integrity. Conversely, the rapid dissociation of test sample B induced observable mucosal injury, despite lower local iron retention. **Conclusions**: These findings suggest that the gastrointestinal tolerability of PICs may be associated with their structural attributes and release behavior, rather than total iron content alone. Overall, this exploratory study highlights a potential relationship between multi-dimensional PIC structure and functional performance, emphasizing the need for broader, structure-informed frameworks in the quality evaluation of complex iron therapies.

## 1. Introduction

Iron deficiency anemia (IDA) remains one of the most prevalent nutritional disorders worldwide and continues to pose a major public health challenge [1,2]. Oral iron supplementation is widely recognized as the first-line treatment because of its effectiveness, accessibility, and relatively low cost [3,4]. However, gastrointestinal (GI) adverse effects, including nausea, abdominal discomfort, and mucosal irritation, frequently compromise patient adherence and may lead to treatment discontinuation [5,6]. Improving the GI tolerability of oral iron formulations therefore remains an important clinical goal.

Polysaccharide–iron complexes (PICs) have been developed as alternatives to conventional inorganic iron salts, with the aim of improving tolerability through controlled iron release [3,7,8]. By coordinating iron within a polymeric matrix, PICs are expected to reduce direct exposure of reactive iron species to the gastrointestinal mucosa [9]. However, the tolerability of PICs is likely to depend on the structural integrity of the complex under gastric conditions [10]. Premature acid-induced disruption of the iron–polysaccharide assembly may lead to the release of free iron, which has been associated with oxidative stress and mucosal irritation [11,12]. Elucidating the structural factors that govern acid stability and iron release is therefore essential for understanding PIC performance.

Unlike small-molecule drugs, PICs are heterogeneous non-biological complex drugs (NBCDs) [13], whose functional properties arise from a combination of higher-order structural attributes rather than a single defined molecular entity [14,15]. Molecular-weight distribution, polysaccharide architecture, and iron coordination microenvironment may collectively influence the stability of the complex under physiological stress. However, the relationship between these structural characteristics and functionally relevant outcomes, particularly under acidic conditions, remains insufficiently understood [14].

At present, the quality evaluation of PICs still relies largely on basic pharmacopeial specifications, such as total elemental iron content and free iron limits. Although these parameters are necessary for routine control, they may not adequately capture functionally meaningful differences in structural organization [16,17]. For NBCDs, even relatively subtle structural variation may translate into differences in release behavior, tolerability, and therapeutic consistency [15,18,19]. Consequently, there is a growing need for function-oriented, multi-dimensional characterization strategies that can better link molecular attributes to clinically relevant outcomes. However, relatively few studies have integrated structural characterization with acid-stress release testing and mucosal-response assessment to investigate functionally relevant differences among commercial oral PIC products. This limits the interpretation of routine quality-control measurements in relation to product performance.

In this study, two commercially available PIC preparations were selected as suitable models for comparative evaluation: a well-established product with international market presence and a regionally available commercial product. They were both marketed as oral PIC products with the same labeled elemental iron content, while differing in physical presentation and formulation design. Molecular-weight distribution, monosaccharide composition, coordination-related spectral features, and polysaccharide-associated structural characteristics were systematically characterized. In vitro dissolution was conducted at pH 1.0 and 1.2 to differentiate the relative stability of the PICs. Although these conditions do not cover the full physiological range of gastric acidity, they provide stringent stress conditions that may reveal release behaviors less evident in milder media. In addition, the physiological relevance of the observed differences was further assessed using an exploratory in vivo model of gastrointestinal tolerability in rats. Crucially, this study aims to generate preliminary mechanistic and quality-assessment evidence to support future evaluation frameworks for complex iron therapies, rather than to establish direct clinical equivalence or definitive clinical safety from these physicochemical and short-term rodent data. By integrating structural characterization with acid-stress release profiling and preclinical biological evaluation, this study aims to provide exploratory evidence for a structure-informed framework for PIC quality assessment, highlighting the technical limitations of relying solely on elemental analysis for NBCDs.

## 2. Materials and Methods

### 2.1. Materials

Two commercial polysaccharide–iron complex capsule products were included in this study. Both contained elemental iron in the form of a polysaccharide–iron complex, equivalent to 150 mg iron per capsule. Test sample A was an imported marketed Niferex capsule containing brown spherical coated pellets. Excipients included a sucrose pellet core, medicated glaze, povidone, and hydrogenated castor oil. Three batches were used: 24279145A, 24279146A, and 22271834A, with expiration dates of April 2027, April 2027, and October 2025, respectively. The product was stored at room temperature (≤25 °C). Test sample B was a locally marketed powder-filled capsule containing a brown to brown-black crystalline powder. Three batches were used: 125027, 125055, and 225054, with expiration dates of January 2028, February 2028, and February 2028, respectively. The product was stored in a tightly sealed container. Excipient information was unavailable. Pullulan standards (22, 110, and 400 kDa) were purchased from Agilent Technologies (Santa Clara, CA, USA). Ferrous ammonium sulfate standard was sourced from National Institutes for Food and Drug Control (NIFDC, Beijing, China). Ferrozine and monosaccharide standards including fucose (Fuc), arabinose (Ara), rhamnose (Rha), galactose (Gal), glucose (Glc), xylose (Xyl), mannose (Man), fructose (Fru), ribose (Rib), galactosamine (GalN), glucosamine (GlcN), galacturonic acid (Gal-UA), glucuronic acid (Glc-UA), mannuronic acid (Man-UA), and guluronic acid (Gul-UA) were obtained from Sigma-Aldrich (St. Louis, MO, USA). 1,10-Phenanthroline was supplied by Adamas-beta (Shanghai, China), and trifluoroacetic acid (TFA) was purchased from ANPEL (Shanghai, China). All other chemicals and solvents were of analytical grade or better and used as received.

### 2.2. Animals

Twenty-eight healthy Sprague–Dawley rats (approximately 200 g, male unless otherwise specified) were purchased from Shanghai Shengchang Biotechnology Co., Ltd. (Shanghai, China). The animals were randomly assigned to seven groups: control, ferrous succinate low dose, ferrous succinate high dose, test sample A low dose, test sample A high dose, test sample B low dose, and test sample B high dose (*n* = 4 per group). Animals were housed under standard laboratory conditions (temperature 22 ± 2 °C, relative humidity 50–60%, and a 12 h light/dark cycle) with free access to standard chow and water. Rats were acclimatized for at least 7 days prior to experimentation. Prior to oral administration, animals were fasted overnight with free access to water. Randomization was performed to assign animals to experimental groups.

### 2.3. Sample Preparation

To minimize interference from insoluble pharmaceutical excipients, commercial PIC capsules were subjected to a mild-extraction procedure prior to structural characterization. Briefly, the capsule contents were dispersed in ultrapure water and centrifuged to remove insoluble fractions. The supernatants were collected and lyophilized to obtain recovered water-soluble PIC fractions. This procedure reduced interference from water-insoluble excipients but may also have removed aggregates or other formulation-associated components. Therefore, the recovered fractions cannot be assumed to represent all components or structural features of the intact commercial products. The resulting powders were stored in a desiccator and used for subsequent structural characterization and monosaccharide composition analysis unless otherwise specified. For assays intended to reflect the performance of the original formulations, including free iron determination and dissolution testing, capsule contents or intact capsules were used directly. Accordingly, the structural characterization and functional assays were performed using different material states, and associations between these results were interpreted cautiously.

### 2.4. Molecular Weight Determination

Number-average molecular weight (*M_n_*), weight-average molecular weight (*M_w_*), and polydispersity index (*M_w_*/*M_n_*, PDI) of the PIC preparations were determined by gel permeation chromatography (GPC). The capsule contents were dissolved in ultrapure water, filtered through an aqueous membrane filter (0.22 μm, which may retain insoluble iron-containing aggregates), and appropriately diluted to a final concentration of approximately 1.125 mg Fe/mL. Chromatographic analysis was performed on an Agilent 1260 Infinity II HPLC system equipped with a refractive index detector (RID) (Agilent Technologies, Santa Clara, CA, USA), using a Waters Ultrahydrogel™ 500 column (7.8 mm × 300 mm, 10 µm) maintained at 35 °C. The mobile phase consisted of 10 mM phosphate buffer (pH 8.0), eluted in isocratic mode at a flow rate of 0.9 mL/min with an injection volume of 20 µL. Molecular weights were calculated based on a calibration curve constructed with pullulan standards ranging from 22 to 400 kDa (R^2^ > 0.99). Because the samples may differ from pullulan standards in hydrodynamic behavior and refractive-index response, the molecular weights reported here are relative values intended for comparative analysis rather than absolute determination.

### 2.5. Mass Spectrometry

Molecular weight distribution of PICs was further qualitatively characterized by matrix-assisted laser desorption/ionization time-of-flight mass spectrometry (MALDI-TOF MS) on a MALDI-7090 instrument (Shimadzu, Kyoto, Japan), operating in positive-ion linear mode. Given the inherent technical challenges of ionizing high-molecular-weight complexes, MALDI-TOF MS was utilized as a complementary technique to support GPC profiles rather than as a definitive quantitative confirmation. Extracted PIC samples (Section 2.3) were prepared using the dried-droplet method with 2,5-dihydroxybenzoic acid (DHB) as the matrix. Acquisition parameters were optimized to obtain representative mass spectra, including a mass range of 15,000–200,000 Da, laser power of 171, and accumulation of 60 to 78 profiles per spectrum. External calibration was performed using a custom calibration method. Data processing, including baseline subtraction and smoothing (smoothing factor = 100), was conducted using the manufacturer-supplied MALDI Solutions software (Shimadzu, Kyoto, Japan), with representative spectra selected from multiple replicate sample spots.

### 2.6. Fourier Transform Infrared (FTIR) Spectroscopy

FTIR spectra were recorded on a Lyza 7000 spectrometer (Anton Paar, Graz, Austria) equipped with an attenuated total reflectance (ATR) accessory. The lyophilized PIC samples (Section 2.3) were analyzed directly. Spectra were acquired over the wavenumber range of 4000–400 cm^−1^ at a resolution of 4 cm^−1^ with 24 replicate scans per sample. Spectral comparisons were performed qualitatively to assess overall coordination complexity rather than using quantitative peak deconvolution.

### 2.7. ^1^H Nuclear Magnetic Resonance (^1^H NMR) Spectroscopy

^1^H NMR spectra were recorded on a Bruker Avance III 600 MHz spectrometer (Bruker BioSpin, Rheinstetten, Germany). Because coordinated iron can cause pronounced paramagnetic relaxation enhancement (PRE) and severe signal broadening, particularly in the anomeric proton region, iron-depleted polysaccharide extracts were additionally prepared to facilitate spectral observation and qualitative comparison. Briefly, PIC samples were heated and stirred in hydrochloric acid solution (pH 1.0) to dissociate the complexes. After centrifugation, the supernatants were concentrated under reduced pressure, and the polysaccharides were selectively precipitated with absolute ethanol. The precipitates were collected by centrifugation and lyophilized. Both intact PIC samples and the corresponding iron-depleted extracts were dissolved in D_2_O and analyzed by ^1^H NMR for comparison. Because the acidic and heated conditions used for iron depletion may hydrolyze glycosidic bonds or otherwise modify the recovered polysaccharide structure, the spectra of the iron-depleted extracts were used only for tentative comparison of signal patterns and were not considered definitive representations of the native polysaccharide backbones.

### 2.8. Monosaccharide Composition Analysis

To determine the detectable monosaccharide profiles under the applied hydrolysis conditions, the recovered water-soluble PIC fractions (2 mg, Section 2.3) were hydrolyzed with 1 mL of 2 M TFA at 121 °C for 2 h. The hydrolysate was evaporated to dryness under nitrogen, and residual TFA was removed by repeated co-evaporation with methanol. The dried residue was reconstituted in ultrapure water and appropriately diluted.

Monosaccharides were analyzed by high-performance anion-exchange chromatography with pulsed amperometric detection (HPAEC-PAD) using an ICS 5000+ system (Thermo Fisher Scientific, Sunnyvale, CA, USA). Separation was achieved on a Dionex CarboPac™ PA20 column (150 × 3.0 mm) maintained at 30 °C. The mobile phases consisted of water (A), 0.1 M sodium hydroxide solution (B), and 0.1 M sodium hydroxide solution containing 0.2 M sodium acetate (C). The flow rate was 0.5 mL/min, and a multi-step gradient elution was applied as follows: 0 min, 95% A/5% B; 26–42 min, 85% A/5% B/10% C; 42.1 min, 60% A/40% C; 52 min, 60% A/40% B; 52.1–60 min, 95% A/5% B for re-equilibration. Quantification was performed via external calibration using a mixture of 15 monosaccharide standards (0.5–20 μg/mL). The reported values represent detectable monosaccharides recovered under the specified TFA hydrolysis conditions and should not be interpreted as the absolute total polysaccharide content of the original product.

### 2.9. Free Iron Content Assay

Free iron content was quantified by UV–Vis spectrophotometry (UV-2600i, Shimadzu, Kyoto, Japan) using the imported drug registration standard for test sample A, which was applied uniformly to both preparations for comparative evaluation. Capsule contents were extracted with sodium acetate buffer (pH 4.5), reduced with freshly prepared ascorbic acid (0.1%, *w*/*v*), and subsequently reacted with ferrozine to form a colored complex. The absorbance was measured at 560 nm. Free iron content was calculated against a ferrous ammonium sulfate standard calibration curve (R^2^ > 0.999) and expressed as a percentage of the labeled iron content. In this study, free iron refers operationally to weakly bound or uncomplexed reactive soluble iron species that are released under mildly acidic conditions (pH 4.5) and reducible by ascorbic acid, rather than strictly pre-existing Fe^2+^.

### 2.10. In Vitro Acid-Stress Dissolution Study

Dissolution tests were performed using USP Apparatus II (paddle method) on a 708-DS dissolution system (Agilent Technologies, Santa Clara, CA, USA). The experiments were conducted at 37 ± 0.5 °C with a paddle rotation speed of 100 rpm. Hydrochloric acid solutions at pH 1.0 and pH 1.2 were used as dissolution media to simulate varying degrees of gastric acid stress. Intact PIC capsules (*n* = 6 per batch) were introduced into 900 mL of dissolution medium. At predetermined time points (15, 30, 45, 60, 90, and 120 min), 10 mL aliquots were withdrawn and replaced with an equal volume of fresh, pre-warmed medium to maintain a constant volume. The withdrawn samples were centrifuged to remove undissolved excipients.

To quantify total released iron, including iron associated with dissolved polysaccharide fragments, 5 mL of the supernatant was subjected to acid digestion (1.5 mL of 1 M sulfuric acid at 60 °C for 45 min). After cooling, ascorbic acid (0.4%, *w*/*v*) was added to reduce iron to the ferrous state. Iron concentration was determined using a 1,10-phenanthroline colorimetric assay, with absorbance measured at 510 nm on a UV–Vis spectrophotometer (UV-2600i, Shimadzu, Kyoto, Japan). Cumulative iron release was calculated based on a ferrous ammonium sulfate calibration curve. To compare dissolution profiles, a two-way ANOVA was performed, followed by Šídák’s multiple comparisons test to evaluate differences at each time point. Differences were considered statistically significant at *p* < 0.05.

### 2.11. In Vivo Gastrointestinal Tolerability Study

To preliminarily evaluate physiological responses, an exploratory in vivo gastrointestinal tolerability study was conducted. Rats were orally administered ferrous succinate (positive control), test sample A, or test sample B at low (15 mg Fe/kg) and high (50 mg Fe/kg) dose levels once daily for seven consecutive days. Each group contained four animals (*n* = 4 per group). A control group receiving vehicle only was included for comparison. The dose levels (15 and 50 mg Fe/kg) were selected to represent a low and high exposure range commonly used in rodent studies of oral iron formulations, enabling evaluation of potential dose-dependent gastrointestinal responses under sub-therapeutic and supra-physiological conditions rather than strict clinical equivalence. During the administration period, body weight and general conditions of the animals were monitored daily. Particular attention was paid to signs potentially associated with gastrointestinal intolerance, including reduced activity, abnormal fecal appearance, diarrhea, or body weight loss.

At the end of the treatment period, animals were euthanized after fasting, and gastrointestinal tissues including stomach and jejunum were collected for histopathological evaluation. Gastric tissue was included to evaluate primary exposure to acidic dissolution conditions, while jejunal tissue was used to assess downstream intestinal effects. Histological evaluation was performed on both gastric and jejunal tissues to comprehensively assess gastrointestinal mucosal responses. Tissue samples were fixed in 10% neutral buffered formalin, embedded in paraffin, sectioned, and stained with hematoxylin and eosin (H&E) for assessment of mucosal integrity and inflammatory alterations.

To further evaluate localized iron accumulation within gastrointestinal tissues, adjacent sections were subjected to Prussian blue staining. Histological injury was evaluated using a semi-quantitative scoring system. Epithelial damage, inflammatory cell infiltration, and structural disruption were each scored on a scale of 0 to 3, where 0 indicated no observable abnormality, 1 indicated mild changes, 2 indicated moderate changes, and 3 indicated severe pathological alterations. All histological assessments were performed in a blinded manner by an experienced pathologist. For each sample, multiple sections were analyzed to reduce sampling bias, and the final score was averaged across sections. If more than one evaluator was involved, inter-observer variability was minimized by consensus scoring. The positive staining area was quantified using ImageJ software (1.8.0.345) and expressed as the percentage of iron-positive area relative to the total tissue area. Histopathological alterations were semi-quantitatively evaluated based on epithelial injury, inflammatory cell infiltration, and structural disruption. All pathological evaluations were performed in a blinded manner. Representative histological fields from both gastric and jejunal sections were selected in a blinded manner to minimize selection bias.

## 3. Results

### 3.1. Molecular Weight Distribution and Structural Heterogeneity

**Figure 1:**
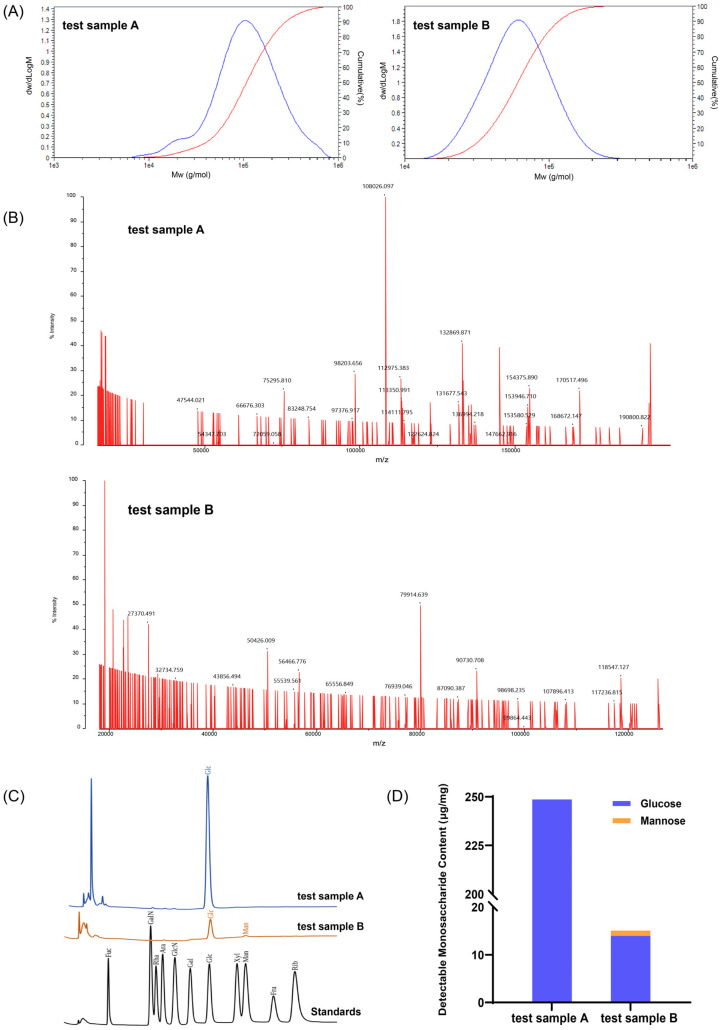
Macromolecular properties and monosaccharide composition of PIC preparations. (**A**) Representative GPC elution profiles illustrating the distinct molecular weight distributions and cumulative fractions of test sample A and B. The blue line represents the molecular weight distribution (dw/dLogM), and the red line represents the cumulative fraction (%). (**B**) MALDI-TOF mass spectra confirming the structural heterogeneity and macromolecular mass of test sample A and B. (**C**) HPAEC-PAD chromatograms comparing the monosaccharide compositions of the two preparations against reference standards. Chromatograms are vertically offset for clarity. (**D**) Stacked bar chart showing detectable monosaccharide content following acid hydrolysis. Test sample A exhibited a markedly higher level of detectable glucose compared to test sample B, whereas mannose was only detected in test sample B.

The apparent molecular weight characteristics of the two PIC preparations were first evaluated by GPC. While limited intra-batch replicates restricted within-batch statistical treatment, the evaluation of three independent batches per preparation provided consistent profile comparisons. Both products fell within the expected pharmacopeial range of 20–200 kDa (Table 1), but exhibited distinct distribution patterns (Figure 1A). Test sample B preparation showed a relatively narrow and symmetric peak with a lower *M_w_* of 69.24 ± 0.35 kDa (PDI = 1.28 ± 0.006), indicating a more homogeneous molecular-weight distribution. In contrast, test sample A preparation displayed a broader distribution with a noticeable shoulder peak, characterized by higher *M_w_* of 134.67 ± 23.14 kDa and a greater PDI of 1.86 ± 0.12 (Welch’s *t*-test, *p* < 0.05), suggesting greater macromolecular heterogeneity.

These findings were further supported by MALDI-TOF MS analysis under DHB matrix conditions (Figure 1B). Test sample B presented a molecular weight distribution primarily in the range of 20–90 kDa, with maximum intensity around 80 kDa. By comparison, test sample A showed a higher mass range of 75–190 kDa with a peak centered around 108 kDa. Both GPC and MS results consistently demonstrate that test sample A possesses a higher molecular weight and a more heterogeneous distribution than test sample B, which may contribute to differences in physicochemical stability and downstream functional performance. Additionally, the *M_w_* of test sample A showed an inter-batch RSD of 17.2%, compared with 0.5% for test sample B, indicating greater numerical variation among the evaluated batches of test sample A. This variability may be associated with the natural origin and polydisperse nature of the polysaccharide precursors, together with the sensitivity of the multi-step iron coordination process.

### 3.2. Monosaccharide Composition and Matrix Characteristics

Monosaccharide composition of the two PIC fractions was analyzed by ion chromatography following acid hydrolysis, revealing distinct differences in both compositional uniformity and detectable sugar content (Figure 1C,D). The polysaccharide matrix of test sample A was composed exclusively of glucose (100%) and exhibited a relatively high level of detectable monosaccharides, reaching 248.61 µg/mg under the applied hydrolysis conditions. In contrast, test sample B displayed a heterogeneous monosaccharide profile, consisting of 92.49% glucose and 7.51% mannose. Notably, the total detectable sugar content in test sample B was markedly lower, with a value of 13.98 µg/mg. This implies that a substantial fraction of its matrix may not be effectively converted into measurable monosaccharides under these specific conditions.

As both PIC samples underwent identical hydrolysis and analytical procedures, the pronounced disparity in sugar recovery may imply differences in polysaccharide accessibility or stability. We hypothesize that this discrepancy potentially results from formulation-related interference, as test sample B may contain unquantified non-carbohydrate, water-soluble excipients, while test sample A may include carbohydrate-based excipients that are more readily detected in the assay. Alternatively, the test sample B matrix might be more susceptible to acid- or iron-catalyzed degradation during hydrolysis, yielding undetectable byproducts. Furthermore, the standardized hydrolysis conditions may not equally efficient for the two matrices, resulting in incomplete depolymerization of test sample B. However, without a comprehensive mass balance or total carbohydrate analysis, the exact cause of this low recovery remains speculative.

### 3.3. Iron Coordination Environment and Polysaccharide Structural Features

**Figure 2:**
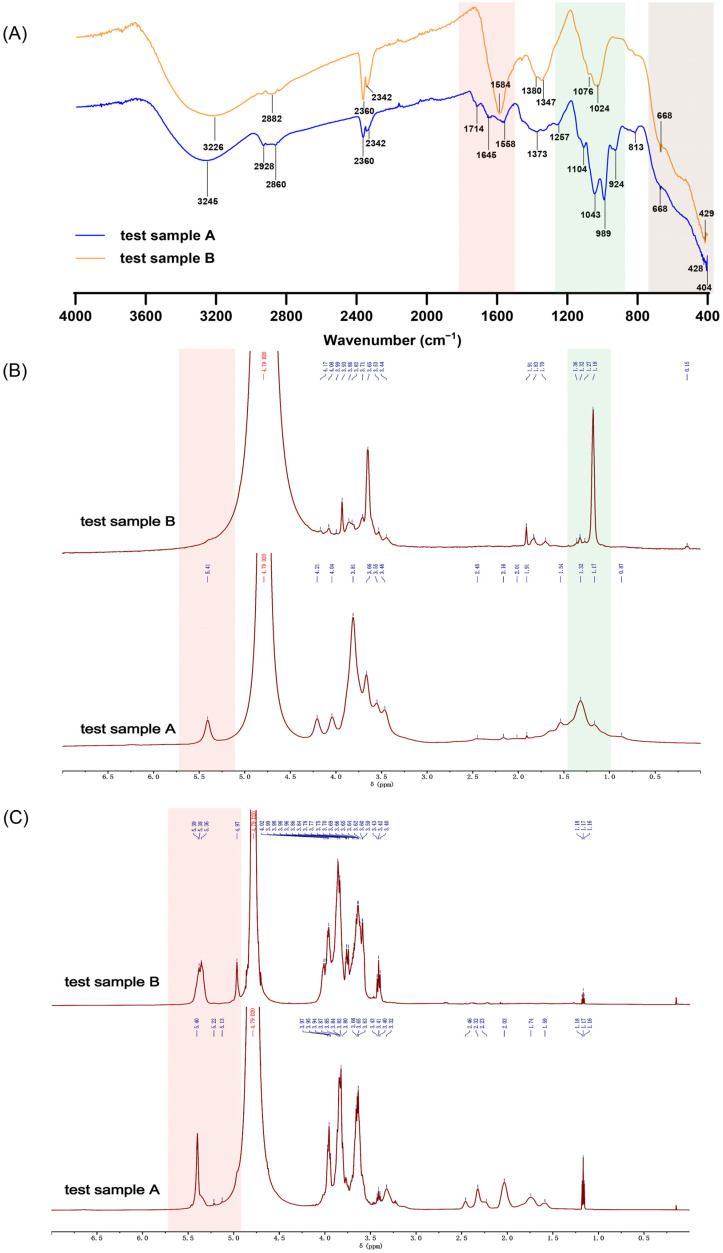
Spectroscopic characterization of PIC preparations and their iron-depleted extracts. (**A**) FTIR spectra showing differences in the spectral features of test sample A and B. The shaded areas indicate the 1800–1500 cm^−1^ region with distinct spectral differences, the 1200–900 cm^−1^ fingerprint region associated with polysaccharide structural features, and the 800–400 cm^−1^ region typically attributed to Fe–O stretching vibrations. (**B**) ^1^H NMR spectra of the intact PIC samples, illustrating the severe signal broadening induced by PRE of the coordinated iron core. The red shaded area indicates the anomeric proton region (δ 5.5–4.9 ppm), whereas the green shaded area indicates the methyl proton region (δ 1.5–1.0 ppm). (**C**) ^1^H NMR spectra of the iron-depleted extracts, showing enhanced signal resolution and altered chemical shift patterns after iron removal. The red shaded area indicates the anomeric proton region, in which differences between the two samples became distinguishable.

FTIR spectroscopy was employed to investigate spectral differences between the recovered PIC fractions (Figure 2A). Both PICs exhibited broad absorption bands between 3200–3400 cm^−1^, characteristic of O–H stretching associated with extensive hydrogen-bonding networks [20]. Distinct differences were observed in 1800–1500 cm^−1^ spectral region. Test sample B showed a relatively sharp and dominant peak at 1584 cm^−1^, whereas test sample A displayed multiple overlapping bands (1714, 1645, and 1558 cm^−1^), indicating differences in the corresponding chemical environments. Although this region may include carbonyl- or carboxylate-associated vibrations [21], contributions from oxidized carbohydrate groups, residual formulation components (such as povidone), or other overlapping vibrations cannot be excluded. Therefore, the origins of these bands cannot be assigned conclusively from the present FTIR data. In the fingerprint region (1200–900 cm^−1^) [22], test sample A exhibited stronger and broader signals compared to test sample B, reflecting differences in polysaccharide structural features. In addition, both PICs showed similar absorption patterns between 800–400 cm^−1^, typically attributed to Fe–O stretching vibrations [23,24]. These spectral differences suggest that test sample A may possess a more intricate coordination environment capable of providing enhanced structural stability.

Further structural insights were obtained from ^1^H NMR analysis. Although the paramagnetic iron core broadened signals [23,25] in both intact samples (Figure 2B), distinct differences emerged in the anomeric proton region. Test sample A showed a relatively sharp signal at 5.4 ppm, characteristic of α-anomeric protons, which suggests the potential presence of α-1,4-glycosidic linkages [26,27], though further 2D NMR or methylation analysis would be required for definitive structural assignment. Conversely, test sample B exhibited weaker and more dispersed signals in this region, which became more distinguishable after partial removal of paramagnetic effects in the iron-depleted extracts (Figure 2C). Signals around 4.97 ppm and multiple miscellaneous peaks at 5.3–5.4 ppm suggest the potential presence of heterogeneous linkages, which might include α-1,6 branching [26,28], though this requires future definitive validation. In addition, signals at 1.1–1.2 ppm were observed in test sample B, attributable to methyl protons. Given the absence of deoxy sugars in the monosaccharide analysis, these signals may arise from residual non-carbohydrate formulation excipients.

Overall, FTIR and ^1^H NMR results consistently suggest that test sample A provides a more intricate coordination environment, whereas the structural signatures of test sample B appear relatively simpler and are potentially influenced by its heterogeneous formulation matrix.

### 3.4. Free Iron Content and Acid-Stress Release Behavior

**Figure 3:**
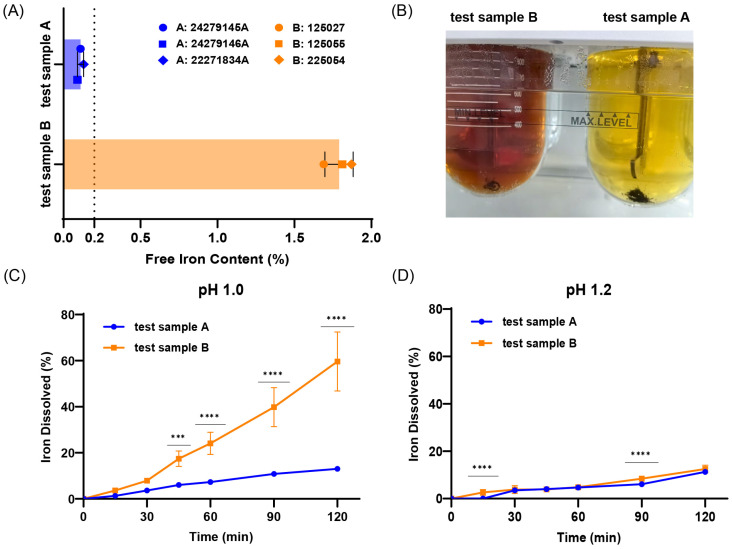
Free iron content and in vitro acid-stress dissolution behaviors of the PIC preparations. (**A**) Free iron content in three batches of test sample A and B. (**B**) Visual appearance of the dissolution media after 120 min at pH 1.0. (**C**,**D**) Cumulative iron release profiles of the PIC capsules under simulated gastric conditions at pH 1.0 (**C**) and pH 1.2 (**D**) over 120 min. Data are presented as mean ± SD (*n* = 6). *** *p* < 0.001, **** *p* < 0.0001. Unmarked comparisons were not statistically significant.

The functional implications of structural differences were further assessed through free iron quantification and in vitro acid-stress dissolution. Free iron contents were determined using a ferrozine-based colorimetric assay (Figure 3A). Test sample A exhibited free iron contents below 0.2% (0.11 ± 0.02%; inter-batch RSD, 18.18%), complying with the specified acceptance criterion. Test sample B showed significantly higher free iron contents (1.79 ± 0.09%; inter-batch RSD, 5.12%) than test sample A (Welch’s *t*-test, *p* < 0.001), suggesting a greater proportion of weakly associated iron species.

Dissolution under simulated gastric conditions further revealed distinct release profiles. At pH 1.0, test sample B showed rapid, highly variable iron release, reaching approximately 60% at 120 min (Figure 3C). As a macroscopic visual observation rather than mechanistic proof of speciation, the test sample B solution developed a deep reddish-brown color (Figure 3B), which was consistent with the elevated total iron release quantified. In contrast, test sample A demonstrated limited release (13%) and remained light-yellow with visible undissolved residues, suggesting preserved macroscopic structural integrity. At pH 1.2, both samples exhibited reduced iron release (Figure 3D). However, test sample B showed pronounced pH-sensitivity with a sharp decrease, while test sample A maintained a consistent, controlled release profile.

Together, these results indicate that test sample A displays greater resistance to acid-induced disruption and more controlled iron release behavior, whereas test sample B is associated with increased acid sensitivity and higher levels of free iron under the tested conditions.

### 3.5. In Vivo Gastrointestinal Tolerability and Mucosal Integrity

**Figure 4:**
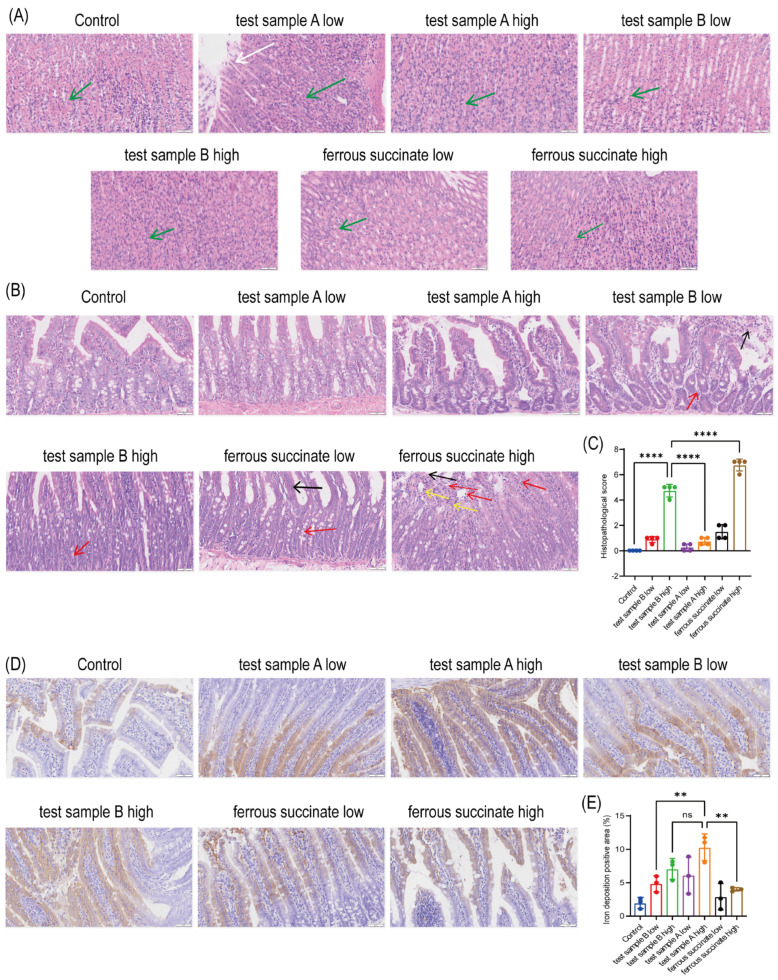
Histological evaluation of gastric and intestinal tissues and intestinal iron deposition analysis. (**A**) Representative hematoxylin and eosin (H&E)-stained gastric sections (20×) from control, ferrous succinate (positive control), test sample A (low/high dose), and test sample B (low/high dose) groups. White arrows indicate gastric pits; green arrows indicate parietal cells. (**B**) Representative H&E-stained jejunal sections (20×) from all experimental groups under identical magnification. Black arrows indicate villus structural damage, red arrows indicate inflammatory infiltration, and yellow arrows indicate stromal-cell-expansion-like changes. (**C**) Semi−quantitative histological injury scores of jejunal tissues across all experimental groups. Data are presented as mean ± SD, with statistical significance indicated in the figure. (**D**) Representative Prussian blue staining of jejunal tissues showing iron deposition in each group. Positive iron signals are indicated by blue staining. (**E**) Quantitative analysis of Prussian blue-positive staining in jejunal tissues across groups, presented as semi-quantitative iron deposition scores. All histological evaluations were performed in a blinded manner. Representative fields were selected from multiple sections to ensure unbiased presentation. All images were acquired under identical magnification (20×) to ensure comparability across groups. Data are presented as mean ± SD (*n* = 4). ** *p* < 0.01, **** *p* < 0.0001, ns, not significant. Scale bars = 50 μm.

To further evaluate the gastrointestinal safety profiles of the two PIC formulations under acid-stress conditions, histological and iron deposition analyses were performed in rats following oral administration. Seven experimental groups were included: control, test sample A (low/high dose), test sample B (low/high dose), and ferrous succinate (low/high dose). Gastric histological examination revealed no obvious structural abnormalities across all experimental groups, including control, test sample A, test sample B, and ferrous succinate groups. Gastric pits and parietal cell architecture remained intact, suggesting that the formulations did not induce detectable gastric mucosal injury under the present experimental conditions (Figure 4A). In jejunal tissues, hematoxylin and eosin (H&E) staining demonstrated that test sample A generally preserved mucosal integrity at both dose levels, whereas test sample B and ferrous succinate groups exhibited more pronounced villus damage and inflammatory cell infiltration, particularly at high doses (Figure 4B). Semi-quantitative histological scoring further confirmed significant differences among groups, with the high-dose test sample B group and ferrous succinate groups showing higher injury scores compared with the control and test sample A groups (*p* < 0.0001) (Figure 4C). Prussian blue staining of jejunal tissues demonstrated iron deposition across all treatment groups. Notably, test sample A exhibited relatively higher iron-positive staining compared with test sample B in Prussian blue-stained sections (Figure 4D). Quantitative analysis further confirmed a higher level of iron-positive area in test sample A compared with test sample B across dose levels (Figure 4E). Overall, these results indicate that both formulation type and dose influence gastrointestinal responses and iron deposition patterns. Importantly, ferrous succinate, used as a conventional oral iron reference, induced more pronounced mucosal injury despite relatively lower and more diffuse iron deposition, highlighting differences in gastrointestinal tolerance among formulations.

Taken together, these findings suggest that mucosal injury is not solely determined by total iron deposition but may also be influenced by formulation-dependent release behavior and local tissue interactions. Ferrous succinate exhibited more pronounced mucosal injury compared with both test sample A and test sample B, serving as a conventional reference for gastrointestinal irritation induced by free iron exposure.

Notably, test sample A showed more pronounced iron deposition than test sample B, particularly under high-dose conditions. However, histological staining alone cannot determine the chemical state, biological activity, or intracellular fate of the deposited iron. Therefore, the observed relationship between iron accumulation and mucosal injury should be interpreted as an association rather than evidence of a protective effect. Test sample A showed greater local iron deposition but relatively preserved mucosal structure, whereas test sample B displayed stronger mucosal responses despite lower iron-positive staining. This discrepancy indicates that gastrointestinal observations do not show a direct correspondence between iron deposition and mucosal injury under the present experimental conditions, but may also depend on the form of iron exposure, acid-triggered release behavior, and local interaction between released iron species and the intestinal mucosa. Together, these in vivo results support the notion that structural differences between PIC formulations, particularly differences in iron coordination stability and acid-release behavior, may lead to distinct intestinal exposure patterns and mucosal tolerability profiles under physiological conditions.

## 4. Discussion

PICs are typical NBCDs, whose performance is closely associated with higher-order structural attributes rather than a single defined molecular entity. Establishing meaningful structure–function relationships is therefore critical for understanding this class of products. In this study, multi-dimensional structural characterization was integrated with acid-stress release profiling and an exploratory in vivo rat tolerability model to compare two commercially available PIC preparations. This approach aimed to explore how structural variations translate into functional disparities affecting gastrointestinal tolerability. The results show that test samples A and B differ substantially in molecular-weight distribution, detectable monosaccharide composition, coordination-related spectral features, free iron content, and acid-response behavior, which may contribute to their distinct mucosal injury profiles in vivo.

Test sample A exhibits higher molecular weight and broader distribution, suggesting a more hierarchical architecture capable of providing enhanced steric shielding. This is complemented by multiple coordination environments revealed by FTIR, indicative of chemically diverse iron-binding modes. In parallel, NMR and monosaccharide analyses support a uniform glucose composition with primary spectral features suggestive of an α-1,4-linked. Drawing upon existing literature, we hypothesize that such α-1,4 linkages could potentially drive the formation of tightly packed helical structures that repel water hydration and proton permeation [29,30,31]. In contrast, test sample B is characterized by lower molecular weight, narrower distribution, simpler coordination signatures, and a compositionally heterogeneous matrix with spectral indications of potential α-1,6 linkages. Although α-1,6 glycosidic linkages are generally considered more stable than α-1,4 linkages at the single-bond level, they tend to promote a more flexible, open, and loosely branched polymer architecture [32,33]. The present results in PIC systems suggest that macroscopic structural organization, rather than intrinsic bond stability alone, may play a more dominant role in determining acid stability. Consequently, it is plausible to hypothesize that a compact and ordered polysaccharide framework could limit water penetration and proton accessibility, whereas a flexible and heterogeneous architecture might facilitate acid-induced disruption [32,34]. Importantly, these findings underscore that structural complexity in PICs is inherently multi-dimensional and cannot be adequately captured by single-parameter specifications.

The functional consequences of these structural differences are reflected in their responses to acid stress. Test sample A maintains low free iron levels and exhibits a controlled, reproducible release under acidic conditions, whereas test sample B shows elevated free iron content and a pronounced pH-sensitive release profile. These observations suggest that test sample A might achieve structural iron retention through a combination of steric shielding from extended polysaccharide chains, chemically diverse coordination, and a potentially more robust matrix network. In contrast, the structural characteristics of test sample B appear to render it more susceptible to acid-induced dissociation. This susceptibility correlates with its rapid iron release and notable pH sensitivity, suggesting a potential for greater variability in physiological environments. From a clinical perspective, human gastric pH is highly dynamic, influenced by food intake, inter-patient variability, and concomitant medications. The strongly pH-dependent release behavior of test sample B suggests that in vivo iron delivery and localized gastric exposure could fluctuate significantly with gastric conditions, which may theoretically increase the risk of gastrointestinal adverse events.

Importantly, in vivo gastrointestinal tolerability study in rats provides a physiological validation of these in vitro findings. H&E histology and Prussian blue staining revealed that test sample A, despite inducing higher local intestinal iron deposition, largely preserved mucosal architecture, whereas test sample B caused more pronounced mucosal alterations even with lower iron accumulation. This dissociation between iron deposition and mucosal injury supports the concept that gastrointestinal tolerability is not solely determined by tissue iron content, but also by the structural form of the iron complex and its acid-triggered release behavior. These in vivo observations reinforce the relevance of multi-dimensional structural attributes in predicting intestinal response and tolerability, highlighting that in vitro acid-stress profiles can provide mechanistic insight into potential in vivo outcomes.

From a regulatory and quality control perspective, the convergence of these structural, in vitro functional, and in vivo tolerability results highlights the potential limitations of conventional specification-based quality assessment for NBCDs. Although different products may meet existing pharmacopeial criteria, their structural and functional behaviors differ substantially, which may affect in vivo performance. As demonstrated, variations in polysaccharide sources, coordination processes, and macroscopic formulation designs can lead to structurally distinct entities with potentially different safety-related attributes. By exploring this structure–function relationship, these findings suggest that the quality and functional consistency of PICs might not be fully captured by simple elemental analysis alone. Therefore, future quality assessments would benefit from incorporating a comprehensive, multi-dimensional framework to better support the therapeutic reliability and safety of complex iron therapies.

Despite providing mechanistic hypotheses, this study is subject to several important limitations. Primarily, although both products are marketed as oral capsules, their internal dosage-form presentations differ: test sample A contains coated pellets, whereas test sample B is formulated as a powder. Therefore, the differences observed in acid-stress release and mucosal tolerability cannot be ascribed solely to the molecular structure of the polysaccharide–iron complexes. Other factors, including pellet disintegration, the protective effect of the coating, and possible excipient-related influences, may also contribute to overall product performance. As a result, the current study design cannot fully disentangle formulation-related effects from the intrinsic properties of the complexes. Furthermore, the in vitro dissolution assay quantified total released iron without performing detailed speciation analysis to differentiate between free Fe^2+^/Fe^3+^, colloidal iron, and complexed soluble iron. In vivo, the animal study was preliminary and exploratory, utilizing a small sample size (*n* = 4 per group) over a short 7-day exposure period, although ferrous succinate was included as a conventional reference to benchmark mucosal injury severity. The absence of biochemical markers precludes a comprehensive evaluation of the spatial tolerability profile. Finally, this study relied on acute mucosal evaluations and did not include long-term pharmacokinetic/pharmacodynamic (PK/PD) assessments or bioavailability data. Future research must utilize standardized, formulation-matched APIs, incorporate quantitative iron speciation and biological oxidative stress markers, and conduct long-term clinical trials to definitively establish how structural heterogeneities influence patient efficacy and safety. This study is limited by the absence of biochemical markers of inflammation and oxidative stress, such as IL-6, TNF-α, MPO, MDA, GSH, and SOD, which would have provided more direct mechanistic insight into iron-induced mucosal responses. Therefore, the current findings are primarily based on histological and morphological observations, and mechanistic interpretations should be considered preliminary.

## 5. Conclusions

This study provides a comparative evaluation of two commercially available PICs using an integrated analytical framework combining structural characterization and functional assessment. Multiple analytical results revealed that test sample A exhibits a uniform monosaccharide composition but a heterogeneous molecular-weight distribution, alongside diverse coordination signatures, which are associated with sustained acid resistance and controlled iron release. Test sample B features a heterogeneous monosaccharide profile but a narrower molecular-weight distribution, coupled with restricted coordination signatures, which may correlate with rapid iron dissociation and highly pH-dependent release under gastric simulation. Consistently, in vivo gastrointestinal evaluation in rats demonstrated that test sample A largely preserved mucosal integrity despite higher local intestinal iron deposition, whereas test sample B induced more pronounced mucosal alterations even with lower iron accumulation.

Overall, these findings highlight that the structural complexity of PICs is inherently multi-dimensional, and their physiological performance may be associated with the interplay of molecular and macroscopic architectural attributes. By integrating structural, functional and in vivo data, this study provides a more comprehensive understanding of the structure–function relationship in PICs. These insights further emphasize the need to move beyond single-parameter specifications toward more holistic, function-oriented evaluation frameworks for NBCDs, incorporating microstructural organization and coordination environments to better predict safety-related outcomes. Nevertheless, the findings of this exploratory study remain preliminary. Further comprehensive investigations are needed to strengthen these proposed structure–function relationships and to inform more robust regulatory evaluation frameworks. Such efforts may include standardized comparative assessments of structure and function that minimize dosage-form effects, validated in situ iron speciation tracking, and larger-scale in vivo pharmacological and toxicological studies.

## Figures and Tables

**Table 1:** GPC results of molecular weight parameters and polydispersity index of the commercial PIC preparations.

Preparation (Batch No.)	*M_w_* (kDa)	*M_n_* (kDa)	PDI (*M_w_*/*M_n_*)
Test sample A (24279145A)	149.59	76.45	1.96
Test sample A (24279146A)	146.40	77.04	1.90
Test sample A (22271834A)	108.01	62.66	1.72
Test sample A	134.67 ± 23.14 *	72.05 ± 8.14	1.86 ± 0.12 *
Test sample B (125027)	69.01	54.06	1.28
Test sample B (125055)	69.64	54.20	1.28
Test sample B (225054)	69.07	53.61	1.29
Test sample B	69.24 ± 0.35	53.96 ± 0.31	1.28 ± 0.006

* *p* < 0.05 compared to test sample B.

## Data Availability

The data presented in this study are available upon request from the corresponding author.
